# A meta-proteomics approach using autopsy material from the pre-antibiotic era from patients with untreated pulmonary tuberculosis to identify proteins present in early lesions of post-primary tuberculosis

**DOI:** 10.1371/journal.pone.0345052

**Published:** 2026-04-01

**Authors:** Beatrice Normann, Syeda Mariam Riaz, Lisbet Sviland, Tehmina Mustafa, Even Birkeland

**Affiliations:** 1 Department of Global Public Health and Primary Care, Centre for International Health, University of Bergen, Bergen, Norway; 2 Department of Pathology, Haukeland University Hospital, Bergen, Norway; 3 Department of Clinical Medicine, Faculty of Medicine, University of Bergen, Bergen, Norway; 4 Department of Thoracic Medicine, Haukeland University Hospital, Bergen, Norway; 5 Department of Biomedicine, Proteomics Unit at University of Bergen (PROBE), University of Bergen, Bergen, Norway; Showa University Fujigaoka Hospital, JAPAN

## Abstract

Primary TB and post-primary TB (PPTB) are different disease entities. PPTB occurs only in humans, and no animal model mimics the actual pathology of PPTB. Information on the immune pathogenesis of PPTB is lacking due to the scarcity of untreated human material. In the early stages of PPTB, lesions can either progress or regress. Mycobacterial proteins present in these early lesions are vital to identifying targets for future development of better vaccines and therapeutics. Using proteomics, we aimed to establish a methodology to identify mycobacterial proteins expressed in lesions of human PPTB from the archival formalin-fixed and paraffin-embedded lung tissue from 1937−1941. Five untreated TB patients with a total of eleven samples were used. Micro-dissected tissue from six early and five necrotic lesions were processed for proteomics. Using a mass spectrometry and multiplexing tandem-mass-tag (TMT) approach, a total of 3531 *Homo sapiens* and 110 bacterial proteins were identified and quantified. Four M. tuberculosis proteins; mIHF (accession p71658), groEL2 (accession P9WPE7), RV2971 (accession P9WQA5), cycA (accession O33203), and 1 *Mycobacterium avium* protein hup (accession A0A0H3A054) were identified. Comparison of early lesions with necrotic lesions showed significantly more RV2971 proteins expressed in early lesions, and mIHF expressed in necrotic lesions (Log2 fold change necrotic – early; 1.401 & −0.581 respectively). In conclusion, we established the methodology and proof of principle for detecting *M. tuberculosis* proteins in PPTB lesions at different stages of the disease. We identified four *M. tuberculosis* proteins, two showing significant differential expression in early and necrotic lesions.

## 1. Introduction

Tuberculosis (TB) is an infectious disease caused by *Mycobacterium tuberculosis* (*M. tuberculosis*), with more than 80% of patients having pulmonary involvement. TB continues to remain a public health priority even after the discovery of effective and affordable antibiotics more than fifty years ago [1]. Primary TB and post-primary TB (PPTB) represent distinct different disease entities. Primary TB develops upon the initial infection with *M. tuberculosis* in individuals who lack prior immunity [2]. In primary TB, infection typically begins with the formation of granulomas, as observed in animal models. The infection begins when the alveolar macrophages phagocytose the inhaled *M. tuberculosis*. Subsequent activation of the adaptive immunity leads to the recruitment of macrophages and lymphocytes at the site of infection, forming an early granuloma, primarily located in the interstitium. The delayed-type hypersensitivity reaction then causes necrosis of immune cells, which manifests morphologically as caseous necrosis at the granuloma center. This immune response is generally effective, controlling primary infection in over 90% of the infected individuals [3]. Latent tuberculosis refers to the presence of M. tuberculosis in the body without clinical signs or symptoms of disease. It may follow primary infection or primary disease, when the host adaptive immune response successfully controls the pathogen and drives it into a dormant state, but rarely eradicates it completely, leading to latent infection.

PPTB represents the host response to a second *M. tuberculosis* infection in individuals who have already developed immunity from a primary infection. Histologically, it presents as a form of tuberculous pneumonia, beginning with an early lesion marked by accumulation of macrophages within the alveolar space [2,4,5]. PPTB occurs only in humans. While it can be studied in certain contemporary animal models, these models do not fully reproduce the histopathological features observed in human PPTB. [4]. This has led to the widespread assumption that granulomas are the defining lesion of all forms of tuberculosis, overshadowing the distinct pathogenesis of PPTB.

The early lesion of PPTB is not a recent discovery; it was extensively characterized both pathologically and radiologically during the pre-antibiotic era. [2,6]. These lesions may progress or regress spontaneously and can even heal without clinical symptoms, as demonstrated by serial X-ray studies used to monitor disease progression in the pre-antibiotic era [7]. These observations indicated that tuberculosis lesions are reversible, as they can undergo absorption. In fact, Dunham et al. reported noticeable absorption in over 12% of TB cases, which was accompanied by clinical improvement [7]. The ability to study these lesions directly with modern immunological tools provides an opportunity to investigate why some lesions progress while others regress. The mycobacterial proteins expressed within these lesions are of particular interest, as they may represent potential vaccine targets for the prevention of PPTB. However, in some lesions, the levels of mycobacterial proteins may be too low to detect.

This study aimed to introduce a novel approach for the detection of low quantities of in vivo expressed mycobacterial proteins in human PPTB lesions. We used laser micro-dissection to dissect lesions at different stages of PPTB disease development. Tissue sections were obtained from the archival formalin-fixed and paraffin-embedded lung tissues from 1937–1941. We used a proteomics approach to detect mycobacterial proteins in the selected lesions and analyzed the differential expression of proteins in early and necrotic lesions.

## 2. Materials and methods

### 2.1. Human autopsy tissue material and preparation of tissue sections

Formalin-fixed paraffin-embedded (FFPE) blocks from 1931–47 were collected from the archives stored at the Department of Pathology, the Gades Institute, Haukeland University Hospital, Bergen, Norway, as a part of another study on 12-02-2019. The inclusion criteria for the earlier study were patients with a diagnosis of TB [8]. TB was diagnosed with conventional diagnosis procedures that included acid-fast staining and culture tests. The clinical information was obtained from the autopsy journals containing all relevant medical history, including patient characteristics, medical history, any procedures or diagnostic tests, diagnosis before death, if any, and autopsy diagnosis, and with a detailed gross and brief microscopic description of autopsy findings. All information was de-identified in accordance with the ethical approval.

FFPE blocks were re-embedded, sectioned using a microtome and stained for haematoxylin and eosin. The TB lesions were identified at different stages of the disease, and TB was confirmed by acid-fast staining.

Sections of 10 micrometres thickness were processed for laser microdissection. The sections were treated with UV light for 10 minutes before mounting onto PEN membrane glass slides and stained manually with haematoxylin-eosin. The lesions of interest were then dissected from the tissue sections using a laser microdissection.

### 2.2. Tissue lysis

The laser microdissected tissue was transferred to 1.5 ml Eppendorf protein low-bind tubes and lysed in 4% SDS, 0.05M TCEP, 0.01M chloroacetamide, and 0.5M TRIS pH 7.6 by heating for 1 hour at 95^°C^ in an Eppendorf shaker at 400 rpm. The samples were then sonicated for 30 seconds, paused for 30 seconds 5 times in a water bath holding at 4^°C^ and repeated three times until the tissue was completely dissolved. Bicinchoninic acid (BCA) assay from Pierce (Thermo Fisher) was used to measure protein concentration of the samples.

### 2.3. Digestion and buffer exchange

Samples were mixed with paramagnetic beads (Sera-Mag Speed beads, GE Healthcare) in a 1:10 ratio. The peptide-bead mixture was then agitated before adding 100% ethanol to make a 70% ethanol solution. Samples were shaken at 1000 rpm in a thermomixer for 7 minutes at room temperature. The reaction mixture was then placed in a magnetic field, causing the paramagnetic beads to attract to a magnet. The supernatant was removed, while the proteins stuck to the beads. The procedure was repeated twice with 80% ethanol to remove the remaining lysis buffer.

50 µl of the digestion buffer containing 100 mM Ammonium biocarbonate/1 mM CaCl_2_ and a trypsin at the concentration of 0.2 µl/µg was added to each sample. The samples were sonicated for 30 seconds and incubated for 16 hours at 37^°C^ in a thermomixer at 1000 rpm. After digestion, the samples were centrifuged at 13,000 rpm at 24^°C^ for 3 minutes. The tubes were placed on the magnetic rack until the beads had settled onto the tube wall before the supernatant was moved to a fresh tube. 50 µl of 0.5M NaCl was added to the remaining beads. The tubes were sonicated for 30 seconds in a water bath and centrifuged at 13,000 rpm at 24°^C^ for 3 minutes. The tubes were placed on the magnetic rack, and when the beads settled onto the tube wall, the supernatant of these tubes was moved to the tube with the previous supernatant. The samples were acidified using 200 µl of 0.1% trifluoroacetic acid (TFA).

### 2.4. Desalting

Oasis 96 well cartridges, Waters, USA, was used for desalting. Cartridges were activated by adding 500 µl of 80% acetonitrile (ACN) with 0.1% formic Acid (FA), centrifuge at 200 x g for 1 minute and the flow through was discarded. Cartridges were washed by adding 500 µl of 0.1% TFA, centrifuged at 200 x g for 1 minute, and the discard was allowed to flow through, and then this step was repeated twice. Samples were added and centrifuged at 100 x g for 3 minutes, and the flow through was discarded. Washed twice with 500 µl of 0.1% TFA, centrifuged at 200 x g for 1 minute. The sample was eluted using 100 µl of 80% ACN with 0.1% FA and centrifuged at 100 x g for 3 minutes. This time, the flow-through was kept. The sample was eluted in 96 well elution plates using 100 µl of 80% ACN with 0.1% FA. Centrifuged at 100 x g for 1 minute, repeated once. The samples were freeze-dried prior to TMT labelling.

### 2.5. TMT labelling

20 µl of 0.5M, 2-(4-(2-hydroxyethyl) piperazine-1-yl) ethane-1-sulfonic acid (HEPES) buffer pH 8.5 was added to each sample, and the protein concentration was measured and adjusted using a Nanodrop (Thermo Fisher). Each TMT label was dissolved in 100% ACN and shaken for 5 minutes at 800 rpm before sonication for 30 seconds. Then, the samples were allowed to rest for 45 minutes, with a vortex every 15 minutes. 15 µl of TMT pro-reagent was added into each sample and then vortexed at 15,000 rpm for 30 seconds. The samples were allowed to rest for 75 minutes. 5 µl of the 5% hydroxylamine in 0.5 M HEPES buffer pH 8.5 was added into each sample before vertexing and then incubated for 15 minutes. The samples were combined. The samples were frozen at −80°^C^ for 20 minutes and freeze-dried in a speed vacuum for 1 hour. Finally, 300 µl of 0.1% TFA was added to the sample tube, shaken at 1500 rpm for 5 minutes, and sonicated for 30 seconds.

### 2.6. Peptide fractionation

Peptides were fractionated into four fractions using the Pierce™ High pH Reversed-Phase Peptide Fractionation Kit. Before mass spectrometry analysis, the eluates were lyophilized and resuspended in 0.5% ACN with 0.01% FA to a one µg/µl concentration.

### 2.7. Mass spectrometry

About 0.5 μg protein as tryptic peptides dissolved in 2% ACN, 0.5% FA, were injected into an Ultimate 3000 RSLC system (Thermo Scientific, Sunnyvale, CA, USA) connected online to Orbitrap Eclipse mass spectrometer (Thermo Scientific) equipped with EASY-spray nano-electrospray ion source (Thermo Scientific). For the trapping and desalting process, the sample was loaded and desalted on a pre-column (Acclaim Pep Map 100, 2 cm × 75 µm ID nano Viper column, packed with 3µm C18 beads) at a flow rate of 5 µL/min for 5 minutes with 0.1% TFA. Peptides were separated during a biphasic ACN gradient from two nanoflow UPLC pumps (flow rate of 250 nL/min) on a 25 cm analytical column (Pep Map RSLC, 50 cm × 75 µm ID EASY-Spray column, packed with 2 µm C18 beads). Solvents A and B were 0.1% FA (vol/vol) in dH_2_O and 100% ACN, respectively. The gradient composition was 5% B during trapping (five minutes), followed by 5–7% B over 30 seconds, 8–22% B for the next 145 minutes, 22–28% B over 16 minutes, and 35–80% B over 15 minutes. Elution of very hydrophobic peptides and conditioning of the column was performed for 15 minutes of isocratic elution with 90% B and 20 minutes of isocratic elution with 5% B, respectively. The peptides eluted from the LC column were ionized in the electrospray and analysed by the Orbitrap Eclipse. The mass spectrometer was operated in the data-dependent-acquisition (DDA)-mode to automatically switch between full scan MS and MS/MS acquisition. Instrument control was through Tune 2.7.0 and Excalibur 4.4.16.14.

A full survey scan of the MS spectrum (from m/z 375–1500) was acquired in the Orbitrap with resolution R = 120,000 at m/z 200 after accumulation to a target value 4e5 in the C-trap. The ion accumulation time was set as auto. FAIMS was enabled using two compensation voltages (CVs), -45V and -65V respectively. The mass spectrometer was operated in DDA mode during each CV to automatically switch between full scan MS and MS/MS acquisition. The cycle time was maintained at 0.9s/CV. The most intense eluting peptides with charge states 2–6 were sequentially isolated to a target value (AGC) of 2e5 and a maximum IT of 120 ms in the C-trap. Isolation width was maintained at 0.7 m/z before fragmentation was performed with normalized collision energy (NCE) of 30%. Fragments were detected in the Orbitrap at a resolution of 30 000 at m/z 200, with the first mass fixed at m/z 110. The spray and ion-source parameters were as follows. Ion spray voltage = 1900 V, no sheath and auliary gas flow, and capillary temperature of 275^°C^.

### 2.8. Data analysis using a curated pulmonary FASTA file database

Raw files were searched against a curated FASTA of 226,274 entries across 25 species in the database (S1 Table in S1 File). The species added to the database were included based on a literature search, where microorganisms known to be related to TB pathology were added together with known pulmonary diseases such as pneumonia, pulmonary diseases and infections, HIV, allergies, and air pollution [9–14]. All the FASTA files were downloaded from Uniprot [15] and combined into one database.

### 2.9. Bioinformatics analysis

Thermo Proteome Discoverer version 2.5 was used to map the MS/MS spectra to the database; for full details, see S1 Fig in S1 File.

Normalisation was performed in Thermo Proteome Discoverer; the abundances were normalised to the same total peptide amount per channel and scaled so that the average abundance per protein and peptide totalled 100. Samples were again normalised in Perseus. Samples 2.2 and 3.1 had a significantly lower total number of identified proteins compared to the other samples, so they were excluded and not further analysed. The data were then transferred to Perseus version 1.6.15.0 [13] for quality check and statistical analysis. S2 and S3 Figs in S1 File show data on mycobacterial protein quality check.

### 2.10. Ethics approval

The project has been approved by the Regional Ethical Committee REK, ref 2019/45. This study uses archival FFPE lung tissue from the same autopsy collection previously described by Riaz et al. [8]. The earlier report focused on histopathological and immunopathological characterization of PPTB lesions. In contrast, the current manuscript presents a new proteomics-based analysis (laser microdissection, TMT-labelling, and mass spectrometry) aimed at identifying and quantifying mycobacterial proteins in early and necrotic PPTB lesions. The proteomic dataset and results were not part of the previous publication.

## 3. Results

There were 81 cases with lung paraffin blocks available. Only 5 cases with easily detectable lesions on polyethylene (PEN) membrane slides were selected for this pilot study. Table 1 shows the characteristics of each patient used in this study.

**Table 1:** Clinical and demographic characteristics of the post primary tuberculosis cases included in the study. Summary of patient information for the five archival PPTB cases used for lesion microdissection and proteomic analysis, including age, gender and cause of death and clinical diagnosis.

Patient	1	2	3	4	5
Year of death	1946	1947	1935	1943	1936
Gender	Male	Male	Male	Female	Male
Age	38 years	45 years	44 years	5 months	20 years
Type of TB	Pulmonary	Pulmonary, extrapulmonary, miliary	Pulmonary	Pulmonary, extrapulmonary, miliary, meningitis	Pulmonary, extrapulmonary, miliary
Cavities	No	No	No	Yes	No
TB as a cause of death	No	Yes	Yes	Yes	Yes
Lesions taken	2 early lesions + 1 necrotic lesion	2 early lesions + 1 necrotic lesion	2 early lesions + 1 necrotic lesion	1 necrotic lesion	1 necrotic lesion

**Fig 1:**
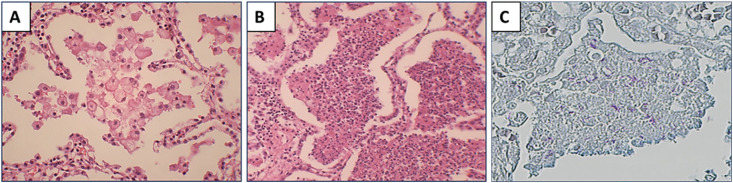
Histopathological definition of lesions used for laser microdissection. **(A)** Haematoxylin and eosin (H&E) stained section showing an early intra-alveolar lesion, defined as an alveolar lesion in which the structure of individual cells can be clearly appreciated. **(B)** H&E stained section showing a necrotic intra-alveolar lesion, defined as an alveolar lesion in which necrosis has started, and the structure of individual cells can no longer be clearly appreciated. **(C)** Acid-fast staining of a necrotic lesion confirming the presence of acid-fast bacilli consistent with mycobacteria.

An early lesion was defined as an intra-alveolar lesion in which the structure of single cells could be appreciated (Fig 1A). A necrotic lesion was defined as an intra-alveolar lesion in which necrosis had started, and an individual cell’s structure could not be appreciated (Fig 1B). This lesion is referred to as a necrotic lesion.

### 3.1. Mycobacterial proteins

**Fig 2:**
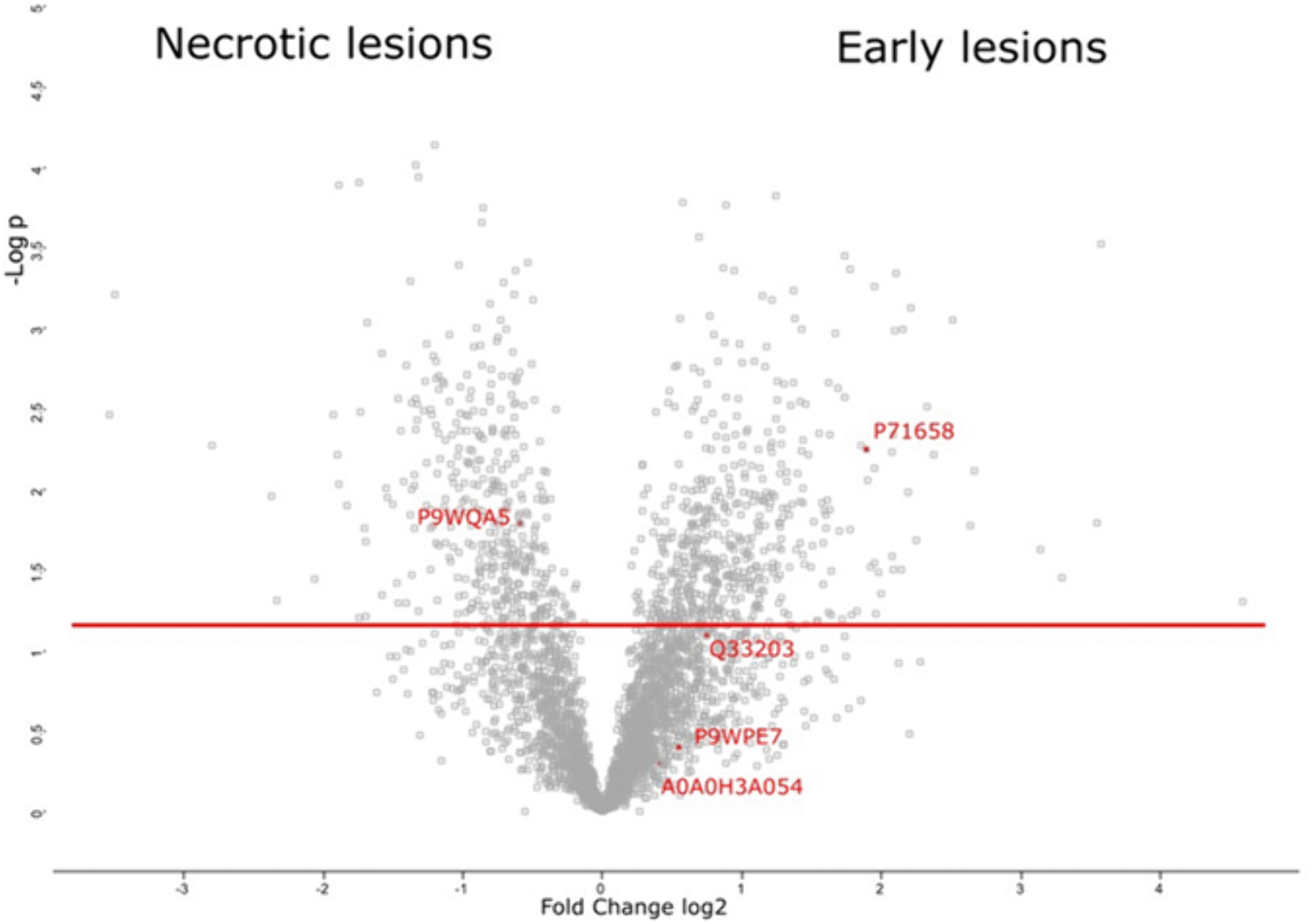
Volcano plot shows differential protein abundance between early and necrotic PPTB lesions.

A total of 3531 *Homo sapiens* and 110 bacterial proteins were identified. S2 Table in S1 File shows the total amount of identified proteins found in each sample, and S3 Table in S1 File shows the certainty of mycobacterial protein identifications and statistical strength through more detailed data from Thermo Protein Discoverer version 2.5. of the identified mycobacterial proteins. Of the 110 bacterial proteins found in the lesions, five were mycobacterial proteins: one belonging to *Mycobacterium avium* and four to *M. tuberculosis*. The Volcano plot in Fig 2 shows the distribution of proteins in early lesions and necrotic lesions. The display shows a clear separation of the proteins found in these two types of lesions. Mycobacterial proteins were scarce among the total proteins and were identified as four *M. tuberculosis* proteins: mIHF, groEL2, RV2971, cycA, and one *M. avium* protein, hup.

Volcano plot summarizing proteins identified by mass spectrometry in laser-microdissected early (n = 6 lesions) and necrotic lesions (n = 5 lesions) from untreated human PPTB. The x-axis shows log2 fold change in protein abundance (necrotic lesion minus early lesion), where positive values indicate proteins enriched in necrotic lesions and negative values indicate proteins enriched in early lesions. The y-axis shows statistical significance as –log10(p-value). Proteins passing the false discovery rate (FDR) threshold of 0.05 are considered significantly differentially abundant. Mycobacterial proteins detected in the dataset are highlighted in red. Statistical analysis and visualization were performed in Perseus.

Volcano plots measure statistical significance using a -log p-value on the *y*-axis and the magnitude of change using a Z-score on the *x*-axis. Each point represents a protein found in the samples.

**Fig 3:**
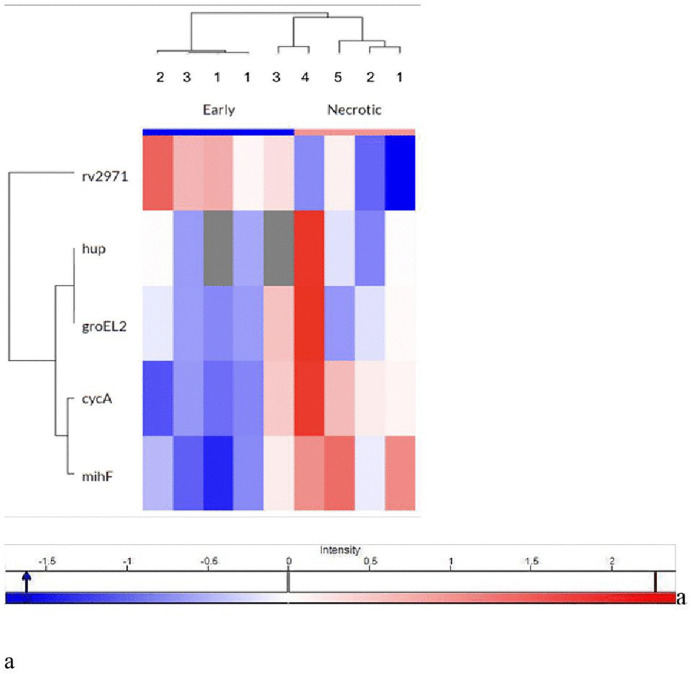
Hierarchical clustering of mycobacterial proteins detected in early and necrotic lesions.

Table 2 describes the five mycobacterial proteins and their comparison in the early and necrotic lesions. RV2971, an aldo-keto reductase of *M. tuberculosis,* was more abundant in early lesions, whereas mIHF, a mycobacterial integration host factor, was more abundant in necrotic lesions (Log2 fold change necrotic – early; 1.401 & −0.581 respectively). There was no difference in the levels of two other mycobacterial proteins: groEL2, a 60 kDa chaperonin 2 protein that prevents aggregation of substrate proteins and promotes their refolding, and cycA, a D-serine/alanine/glycine transporter protein. Hup B, a DNA-binding protein of *M. avium,* was not differentially expressed in the two types of lesions (Table 2, Fig 3).

**Table 2:** Mycobacterial proteins detected in early and necrotic post primary tuberculosis lesions. List of mycobacterial proteins identified by MS-based proteomics in laser-microdissected early and necrotic lesions, including accession number, gene name, species assignment, and protein description. Differential abundance is shown as log2 fold change (necrotic − early): positive values indicate higher abundance in necrotic lesions, and negative values indicate higher abundance in early lesions.

Accession	Gene	Bacteria	Description	Log2 fold change (necrotic – early)
*P71658	mIHF	*Mycobacterium tuberculosis* (strain ATCC 25,618/H37Rv)	Mycobacterial integration host factor (mIHF).	1.401
P9WPE7	groEL2	*Mycobacterium tuberculosis* (strain ATCC 25,618/H37Rv)	60 kDa chaperonin 2; prevents aggregation of substrate proteins and promotes their refolding	0.561
A0A0H3A054	hup	*Mycobacterium avium* (strain 104)	Hup B – DNA-binding protein	0.825
*P9WQA5	Rv2971	*Mycobacterium tuberculosis* (strain ATCC 25,618/H37Rv)	Aldo-keto reductase	− 0.581
O33203	cycA	*Mycobacterium tuberculosis* (strain ATCC 25,618/H37Rv)	CycA - D-serine/alanine/glycine transporter protein	0.778

Supervised hierarchical clustering heatmap showing relative abundance of mycobacterial proteins identified in microdissected lung lesions (early lesions, n = 6; necrotic lesions, n = 5). Protein abundance values were log2-transformed and displayed as z-scores (row-wise normalization), where higher z-scores represent higher relative abundance of a protein across samples. Each column represents an individual lesion sample, and each row represents a detected mycobacterial protein. Clustering was performed in Perseus, and the color scale indicates relative abundance (z-score).

### 3.2. Other bacterial and fungal proteins

**Fig 4:**
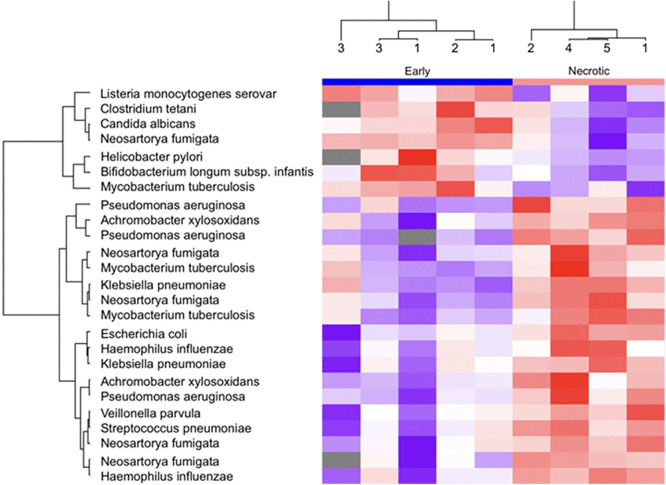
Hierarchical clustering of significantly differentially abundant bacterial and fungal proteins between lesion stages.

In total, 105 microbial proteins from 25 microbial species other than mycobacteria were identified. Table 3 lists the different fungal and bacterial species and the number of their identified proteins. From these proteins, 25 were significantly differentially expressed between early and necrotic lesions, as shown by hierarchical clustering in Fig 4. Seven bacterial proteins were found in the early lesions more than the necrotic lesions, while 18 were found in the necrotic lesions more than the early lesions. Table 4 shows the description of proteins. Four of the abundant seven proteins in the early lesions were found only in early lesions. These proteins were HP_1024 from *Helicobacter pylori,* lmo0282 from *Listeria monocytogenes senvar*, CRN1 from *Candida albicans*, and Blon_2093 from *Bifidobacterium longum subsp. Infantis*. Candida albicans and Neosartorya fumigata were the most abundant fungal proteins found in the TB lesions. Candida albicans was significantly more in early lesions.

**Table 3:** Number of identified proteins from the various bacterial and fungal species in early and necrotic lesions of post-primary pulmonary TB.

Bacteria	Number of identified proteins
*Neosartorya fumigata*	19
*Candida albicans*	14
*Burkholderia cenocepacia*	7
*Escherichia coli*	7
*Pseudomonas aeruginosa*	7
*Achromobacter xylosoxidans*	6
*Clostridium tetani*	4
*Klebsiella pneumoniae*	4
*Mycobacterium tuberculosis*	4
*Helicobacter pylori*	3
*Listeria monocytogenes serotype 1/2a*	3
*Neisseria meningitidis serogroup A/serotype 4A*	3
*Neisseria meningitidis serogroup B*	3
*Staphylococcus aureus*	3
*Veillonella parvula*	3
*Bacteroides fragilis*	2
*Bacteroides thetaiotaomicron*	2
*Bifidobacterium longum subsp. Infantis*	2
*Haemophilus influenzae*	2
*Moraxella catarrhalis*	2
*Prevotella ruminicola*	2
*Streptococcus pneumoniae*	2
*Streptococcus pneumoniae serotype 4*	2
*Enterobacteriaceae bacterium*	1
*Helicobacter hepaticus*	1
*Mycobacterium avium*	1
*Ureaplasma parvum serovar 3*	1

**Table 4:** Significantly differentially expressed bacterial and fungal proteins in early and necrotic pulmonary tuberculosis lesions, including their description and Log2 fold change.

Accession	Gene	Bacteria	Description	Log2 fold change (necrotic – early)
A0A0H2V898	c2405	*Escherichia coli*	Uncharacterised protein	3.138
A0A1D8PQZ0	CRN1	*Candida albicans*	Coronin	−0.765
A4D9K1	AFUA_1G08795	*Neosartorya fumigata*	Shugoshin family protein	0.808
A6TCD1	yfgL	*Klebsiella pneumoniae*	Outer membrane protein assembly factor BamB	0.505
A6TCR1	KPN_02976	*Klebsiella pneumoniae*	Putative bacteria extracellular solute-binding protein, family 3	1.046
A6V4 × 5	PSPA7_2750	*Pseudomonas aeruginosa*	Putative transcriptional regulator	1.776
B7GUL0	Blon_2093	*Bifidobacterium longum subsp. infantis*	DUF4365 domain-containing protein	−2.066
D1BM10	Vpar_0736	*Veillonella parvula*	SMI1_KNR4 domain-containing protein	2.142
E3HFI1	AXYL_01937	*Achromobacter xylosoxidans*	Uncharacterised protein	0.661
E3HMD8	AXYL_03696	*Achromobacter xylosoxidans*	ABC transporter family protein	0.670
O25668	HP_1024	*Helicobacter pylori*	Co-chaperone-curved DNA binding protein A (CbpA)	−2.334
O33203	cycA	*Mycobacterium tuberculosis*	Probable D-Serine/alanine/glycine transporter protein CycA	0.778
P44304	gapA	*Haemophilus influenzae*	Glyceraldehyde-3-phosphate dehydrogenase	0.667
P44909	pdxH	*Haemophilus influenzae*	Pyridoxine/pyridoxamine 5’-phosphate oxidase	1.509
P71658	mIHF	*Mycobacterium tuberculosis*	Purative interrogation host factor mIHF	1.401
P9WQA5	Rv2971	*Mycobacterium tuberculosis*	Uncharacterised oxidoreductase Rv2971	−0.581
Q4WHG5	lia1	*Neosartorya fumigata*	Deoxyhypusine hydroxylase	0.933
Q4WJ30	AFUA_1G07440	*Neosartorya fumigata*	Molecular chaperone Hsp70	0.287
Q4WWR1	AFUA_3G06760	*Neosartorya fumigata*	60S ribosomal protein L37	−2.375
Q4 × 0D4	AFUA_2G13860	*Neosartorya fumigata*	Histone H4	1.009
Q898G4	CTC_00488	*Clostridium tetani*	Conserved protein	−1.229
Q8YA77	lmo0282	*Listeria monocytogenes serovar*	Lmo0282 protein	−0.854
Q97NX6	scpB	*Streptococcus pneumoniae*	Segregation and condensation protein B	1.120
Q9HZI6	PA3020	*Pseudomonas aeruginosa*	Probable soluble lytic transglycosylase	1.026
Q9I479	PA1265	*Pseudomonas aeruginosa*	Uncharacterised protein	0.860

Supervised hierarchical clustering heatmap of microbial proteins (bacterial and fungal, excluding mycobacteria) that were significantly differentially abundant between early (n = 6) and necrotic (n = 5) PPTB lesions. Abundance intensities were log2-transformed and displayed as z-scores (row-wise normalization) to enable comparison across samples. Proteins shown passed an FDR threshold of 0.05. Each column represents an individual lesion sample, and each row represents a microbial protein. Visualization and clustering were performed in Perseus, and the color scale represents relative protein abundance across samples (z-score).

## 4. Discussion

Using the proteomics-based approach, we analyzed the proteins present in early and necrotic lesions of human PPTB from archival formalin-fixed and paraffin-embedded lung tissues collected between 1931–1947. In total, 3,531 human proteins and 110 bacterial proteins were identified, including five of mycobacterial origin, one from *Mycobacterium avium* and four from *M. tuberculosis*. *M. tuberculosis* proteins identified were differentially expressed in early and necrotic lesions of PPTB.

Recent proteomics studies have highlighted the clear spatial and temporal heterogeneity of TB lesions, particularly within granulomas, demonstrating that host and pathogen protein expression varies significantly across lesion microenvironment [13,16,17]. Laser-capture microdissection–based proteomics analysis of human TB tissue has primarily focused on host immune and inflammatory pathways, with limited detection of bacterial proteins due to their low abundance relative to host proteins [16]. In this context, our study complements previous work by demonstrating the feasibility of detecting in vivo–expressed mycobacterial proteins directly within different lesions of untreated human PPTB using archival FFPE material.

One protein of interest, RV2971, was found to be more abundant in early than necrotic lesions. RV2971 is an Aldo-Keto Reductase (AKR) of *M. tuberculosis* and has been identified as a target of isoniazid, a key first-line drug against TB. Isoniazid acts by inhibiting cell-wall synthesis in *M. tuberculosis* [18]. The AKR is widely distributed among microorganisms and includes enzymes that catalyze redox reactions essential for biosynthesis, intermediary metabolism, and detoxification [19]. *M. tuberculosis* only has two putative AKRs, Rv2971 and Rv2298. Among these, Rv2971 has been characterized as an essential gene for bacterial growth and survival, functioning in the detoxification of methylglyoxal, a cytotoxic metabolite that can induce DNA damage and trigger apoptosis. Previous proteomic studies in experimental TB models and human lesions have shown that active early lesions are enriched for bacterial proteins involved in redox homeostasis, stress response, and intracellular survival, supporting the biological relevance of increased Rv2971 abundance in early PPTB lesions [13,17]. The first-line drug isoniazid exerts its activity by inhibiting cell-wall synthesis. Studies have also shown that *M. tuberculosis* evades the host immune response by modulating apoptotic pathways, including the overexpression of FASL on macrophages [20–22], thereby exploiting macrophages as a niche for its survival. The elevated expression of AKR observed in early lesions of human PPTB is consistent with and further supports these findings.

The mIHF protein was found to be significantly more abundant in necrotic lesions. mIHF is one of the nucleoid-associated proteins (NAPs) of *M. tuberculosis*. Only four NAPs have been identified in the *M. tuberculosis* genome: *EspR*, *HupB*, *Lsr2* and *mihF*. The gene *mihF* is highly conserved across the Mycobacterium genus, and even *Mycobacterium leprae*, despite its greatly reduced genome, retains a copy of *mihF* (*ihf10*) [23]. NAPs are abundant, low-molecular- weight polypeptides that bind to DNA, altering its conformation and thereby modulating transcription. In addition, some NAPs can also bind RNA, influencing the gene expression profile of the cell at a post-transcriptional level [24]. Proteomic studies have shown NAPs are essential for bacterial persistence, stress tolerance, and survival within hostile host environments, particularly under conditions associated with hypoxia and necrosis [23,25]. The mIHF protein is highly stable and exclusively cytosolic. It is essential for growth and survival, influencing both protein and nucleic acid synthesis [25]. It exerts a pleiotropic effect on gene regulation in *M. tuberculosis* by controlling the expression of housekeeping as well as virulence genes. Notably, protein depletion led to dramatic changes in cell morphology and global physiology [23]. Its upregulated expression in necrotic lesions suggests that mIHF could be a promising target for adjuvant therapies, where modulation of its expression may help limit tissue destruction, thereby reducing cavity formation and disease transmission.

The proteins groEL2 and cycA were detected in both early and necrotic lesions but showed no differential expression. GroEL2 belongs to the chaperonin family of heat shock proteins. which are widely distributed among bacteria and function to prevent protein misfolding under stress conditions, such as elevated temperature [26]. *M. tuberculosis* encodes two chaperonin genes, groEL1 and groEL2; of which only groEL2 is essential. GroEL2 is an abundant protein linked to virulence [27], localized in both the cytoplasm and the outer cell wall layer. Proteomics studies on human and animal TB models have consistently identified GroEL2 as a dominant bacterial protein involved in immune modulation, apoptosis inhibition, and macrophage survival, supporting its detection in lesion of PPTB [28,29]. It is critical for mycobacterial survival and has even been detected in the cerebrospinal fluid of patients with TB meningitis [30]. Unlike typical chaperonins, GroEL2 functions as a dimer and lacks ATPase activity. Instead, it is highly antigenic, capable of stimulating the release of interleukin-10 and tumor necrosis factor-alpha from monocytes in a CD14-independent manner [31]. It also contributes to *M. tuberculosis* phagocytosis through its interaction with the macrophage surface molecule CD43 [32]. GroEL2 has also been shown to modulate dendritic cell function. The full-length protein elicits strong proinflammatory activity, driving dendritic cell maturation and enhancing antigen presentation to T cells. In contrast, the cleaved form of GroEL2, which predominates in *M. tuberculosis*, induces a weak immune response and fails to promote dendritic cell maturation and antigen presentation, thereby delaying the onset of antigen-specific T-cell responses [29]. GroEL2 also exhibits strong anti-apoptotic activity, which depends on its interaction with host mitochondrial protein mortalin. It detaches from the *M. tuberculosis* surface, crosses the phagosomal membrane, and localizes to macrophage mitochondria, where it interacts with mortalin, a member of the HSP 70 gene family, involved with apoptosis regulation, thereby blocking apoptosis of *M. tuberculosis* infected macrophages [28]. Successful establishment of *M. tuberculosis* relies on its ability to inhibit apoptosis in macrophages [33]. The detection of groEL2 in PPTB lesions supports earlier findings that *M. tuberculosis* evades innate immunity by interfering with macrophage apoptosis [20–22,34]. By doing so, the bacterium exploits macrophages as a protective niche for survival, where MTB antigens accumulate until the cells eventually undergo necrosis [20–22,34].

CycA is a D-serine, L-alanine, and glycine transporter protein in *M. tuberculosis*. In addition to its physiological role, it also mediates the uptake of D-cycloserine, an important second-line anti-tuberculosis drug. Notably, overexpression of D-alanine has been shown to increase resistance to D-cycloserine [35,36]. Both L- and D-alanine are essential for *M. tuberculosis* growth, as they are required for the synthesis of peptidoglycan in the cell wall. Peptidoglycan not only protects and stabilizes the bacterial cell but also serves as a critical regulator of cell division, making it indispensable for *M. tuberculosis* survival and proliferation [37]. Thus, CycA contributes to *M. tuberculosis* survival and holds potential as a biomarker for TB, useful for monitoring host responses to treatment. Previous TB proteomics studies have detected amino acid transporters and cell wall–associated proteins across multiple lesion types, underscoring their fundamental role in bacterial viability rather than stage-specific pathology, consistent with our findings [13,16].

Human tissue remains the only truly relevant source for studying the immunopathogenesis of PPTB, since this form of the disease occurs exclusively in humans. Animal models, including non-human primates, are limited to replicating primary TB. There is a shortage of human TB material for research purposes. Following the introduction of antibiotics and the resulting decline in TB-related mortality, such material became increasingly difficult to obtain. Moreover, most post-antibiotic era patients have undergone treatment, and the protein composition of their lesion samples may differ from that of untreated cases in the pre-antibiotic era. Moreover, few laboratories have systematically preserved historical autopsy material. Recent lesion-focused proteomics studies have emphasized the importance of analyzing human tissue to capture disease-specific mechanisms that are not reproduced in experimental systems, highlighting the value of archival collections such as the Norwegian TB material used here [13,16]. Because of this scarcity, researchers have had to rely on animal models, which differ in their pathogenesis from human disease. The predominance of studies on primary TB over PPTB has created a significant knowledge gap in understanding the latter. The Norwegian archival TB material is well-preserved and suitable for advanced immunological and microbiological analyses. In addition to autopsy samples, medical and epidemiological records in many countries represent unique resources. Together, these materials make it possible to investigate relevant human samples with modern techniques, offering new and invaluable insights into the immunopathology of PPTB.

Laser microdissection is a technique that enables the analysis of specific cell populations within the context of heterogeneous tissue morphology. The method allows specific cells to be dissected from various tissue samples, which are then available for further analytical techniques. Proteomic analysis can provide information about protein expression as a function of time and microenvironment. It helps unravel disease mechanisms and cellular functions essential for biomarker discovery [38–41].

The cases were carefully selected for the microdissection of lesions, as it was crucial to correctly identify and laser-dissect early and necrotic lesions on PEN slides. Only alveolar lesions were collected to avoid an overwhelming presence of host tissue proteins hindering the detection of bacterial proteins. The study was aimed at establishing proof of principle. However, the lack of statistical significance due to insufficient samples makes generalizing difficult. Nevertheless, by grouping lesions into early and necrotic, we were able to examine differential protein expressions and identify several proteins of interest. Importantly, this study achieved its primary goal of establishing a methodology for detecting *M. tuberculosis* proteins in PPTB lesions across different stages of disease.

Mass spectrometry-based proteomics using FFPE tissue samples typically results in lower proteome coverage compared to fresh frozen tissue due to crosslinking. FFPE tissue generally shows a 10–15% reduction in proteome coverage relative to fresh frozen samples. Although this represents a significant reduction in proteome coverage, proteomics of FFPE samples remain highly valuable because of the large number of available samples, long follow-up times, and detailed metadata.

### 4.1. Limitations and generalizability

The limitation of this study is the small number of patients included (n = 5), and the limited number of lesions analyzed (six early lesions and five necrotic lesions). This work was intended as proof of principle, and the limited sample size reduces the statistical strength of the comparisons and increases the effect of variation between cases. TB lesions are heterogeneous, and differences in lesion microenvironment, disease stage, and tissue preservation may affect protein recovery and detection sensitivity. Therefore, the differential expression observed for individual mycobacterial proteins, including Rv2971 and mIHF, should be interpreted with caution, and extrapolation of these findings to broader TB populations is limited. In addition, the low abundance of bacterial proteins relative to host proteins, particularly in early lesions, may have restricted detection of additional mycobacterial proteins that could be relevant for disease progression or regression. Future studies including larger numbers of untreated human cases and systematic sampling of a greater number of lesions at defined stages will be required to validate the findings and determine whether the identified proteins represent consistent stage-associated mycobacterial expression patterns in PPTB.

## 5. Conclusions

By using microdissection, TMT-labelling and mass spectrometry, we identified 3531 human,110 bacterial and fungal proteins from 80-year-old archival formalin-fixed and paraffin-embedded lung tissues of TB patients. Notably, five mycobacterial proteins were detected, two of which showed significant differential expression: Rv2971, enriched in early lesions, and mIHF, enriched in necrotic lesions of PPTB.

Proteomics offers a powerful approach for advancing our understanding of TB pathogenesis. This pilot study demonstrates the feasibility of detecting mycobacterial proteins amidst the overwhelming abundance of host protein. These proteins would otherwise remain undetected using conventional methods. By enabling the identification of key *M. tuberculosis* proteins expressed at different disease stages and involved in immune modulation and virulence, this approach provides valuable insights that may inform the development of improved diagnostics, vaccines, and therapies for TB.

## Supporting information

S1 File S1 Fig. Proteome Discoverer workflow and key settings used for peptide identification and TMT-based quantification. (A) Processing workflow used to map MS/MS spectra to the curated FASTA database, including peptide-spectrum matching, target/decoy strategy, and false discovery rate (FDR) filtering to assign peptide and protein confidence. (B) Consensus workflow used for reporter-ion quantification, normalization, and scaling across samples. Key workflow parameters are provided in the supplementary methods section. S2 Fig. Manual validation of bacterial peptide identification using MS1 precursor isotope pattern and MS2 fragmentation. Representative MS1 precursor isotope pattern illustrating manual interrogation of mass spectra for bacterial proteins. Multiple candidate peptide matches are shown; the peptide selected by Proteome Discoverer as the highest-confidence match (highlighted in yellow) was subsequently fragmented for MS2 confirmation. The y-axis indicates signal intensity (×10³), and the x-axis indicates mass-to-charge ratio (m/z). Spectra were visualized using Thermo Proteome Discoverer v2.5. S3 Fig. Precursor isotope patterns and interference levels for mycobacterial peptide identifications across samples. MS1 precursor isotope patterns for the peptides used to identify mycobacterial proteins in each sample. In all cases, a single peptide supported protein identification. Sample P71658 showed the highest isolation interference, consistent with multiple potential peptides matches; however, Proteome Discoverer resolved a single high-confidence peptide identification. S1 Table. Curated FASTA database composition used for metaproteomic MS/MS searches. List of species included in the custom reference database used for peptide-spectrum matching, including organism name, UniProt proteome identifier, number of proteins (proteomes) included, and rationale for inclusion based on known associations with TB pathology and pulmonary comorbidities. S2 Table. Total number of proteins identified per sample. Total protein identifications obtained per sample after database search and filtering, grouped by lesion type (early versus necrotic). S3 Table. Quality metrics for mycobacterial protein identifications. Detailed identification and confidence metrics for mycobacterial proteins detected in PPTB lesions, including accession number, gene name, confidence assignment, peptide-spectrum match (PSM) ambiguity, number of unique peptides, isolation interference (%), and Percolator statistics (q-value and posterior error probability [PEP]). For proteins measured in duplicate MS runs, multiple values are reported.
(ZIP)

## Abbreviations

TB: Tuberculosis
M. tuberculosis: *Mycobacterium tuberculosis*
FFPE: Formalin-fixed paraffin-embedded
MS/MS: Tandem mass spectrometry
m/z: Mass-to-charge ratio
H2O: Water
TCEP: Tris(2-carbocyethyl) phosphine
CAA: Chloroacetamide
BCA: Bicinchoninic acid
AmBic: Ammonium bicarbonate
CaCl2: Calcium chloride
HCL: Hydrochloric acid
NaCl: Sodium chloride
TFA: Trifluoracetic
ACN: acetonitrile
HE: Haematoxylin eosin
emPAI: Exponentially modified protein abundance index
PSM: Peptide-spectrum match
