# Supplementary material for: A meta-proteomics approach using autopsy material from the pre-antibiotic era from patients with untreated pulmonary tuberculosis to identify proteins present in early lesions of post-primary tuberculosis
Source: PLoS One. 2026 Apr 1;21(4):e0345052. doi: 10.1371/journal.pone.0345052 (PMC13042780; doi:10.1371/journal.pone.0345052)
Supplement: S1 File — (A) Processing workflow used to map MS/MS spectra to the curated FASTA database, including peptide-spectrum matching, target/decoy strategy, and false discovery rate (FDR) filtering to assign peptide and protein confidence. (B) Consensus workflow used for reporter-ion quantification, normalization, and scaling across samples. Key workflow parameters are provided in the supplementary methods section. S2 Fig. Manual validation of bacterial peptide identification using MS1 precursor isotope pattern and MS2 fragmentation. Representative MS1 precursor isotope pattern illustrating manual interrogation of mass spectra for bacterial proteins. Multiple candidate peptide matches are shown; the peptide selected by Proteome Discoverer as the highest-confidence match (highlighted in yellow) was subsequently fragmented for MS2 confirmation. The y-axis indicates signal intensity (×10³), and the x-axis indicates mass-to-charge ratio (m/z). Spectra were visualized using Thermo Proteome Discoverer v2.5. S3 Fig. Precursor isotope patterns and interference levels for mycobacterial peptide identifications across samples. MS1 precursor isotope patterns for the peptides used to identify mycobacterial proteins in each sample. In all cases, a single peptide supported protein identification. Sample P71658 showed the highest isolation interference, consistent with multiple potential peptides matches; however, Proteome Discoverer resolved a single high-confidence peptide identification. S1 Table. Curated FASTA database composition used for metaproteomic MS/MS searches. List of species included in the custom reference database used for peptide-spectrum matching, including organism name, UniProt proteome identifier, number of proteins (proteomes) included, and rationale for inclusion based on known associations with TB pathology and pulmonary comorbidities. S2 Table. Total number of proteins identified per sample. Total protein identifications obtained per sample after database search and filter [file pone.0345052.s001.zip › S3_Table.docx]

**Supplementary Table 3: Quality metrics for mycobacterial protein identifications.** Detailed identification and confidence metrics for mycobacterial proteins detected in PPTB lesions, including accession number, gene name, confidence assignment, peptide-spectrum match (PSM) ambiguity, number of unique peptides, isolation interference (%), and Percolator statistics (q-value and posterior error probability [PEP]). For proteins measured in duplicate MS runs, multiple values are reported.

| Accession | Gene | Confidence | PSM ambiguity | Number of peptides | Isolation interference (%) | Percolator q-value | Percolator PEP |
| --- | --- | --- | --- | --- | --- | --- | --- |
| P71658 | mihF | High | Unambiguous | 1* | 86 | 0.006 | 0.047 |
| P9WPE7 | groEL2 | High | Unambiguous | 2* | 19 | 0.000039 | 0.00011 |
| P9WPE7 | groEL2 | High | Unambiguous | 2* | 11 | 0.00011 | 0.00035 |
| P9WPE7 | groEL2 | High | Unambiguous | 2* | 21 | 0.00011 | 0.00058 |
| A0A0H3A054 | hup | High | Unambiguous | 1* | 36 | 0.0011 | 0.011 |
| A0A0H3A054 | hup | High | Unambiguous | 1* | 49 | 0.0023 | 0.022 |
| P9WQA5 | Rv2971 | High | Unambiguous | 1* | 20 | 0.001 | 0.01 |
| P9WQA5 | Rv2971 | High | Unambiguous | 1* | 12 | 0.0014 | 0.015 |
| P9WQA5 | Rv2971 | High | Unambiguous | 1* | 31 | 0.0035 | 0.03 |
| O33203 | cycA | High | Unambiguous | 1* | 8 | 0.0014 | 0.015 |

Abbreviations: PSM, peptide spectrum matches; PEP, posterior error probability; *, unique peptides. All proteins were found to have a high confidence and the PSM is the only match as there is no ambiguity. These proteins also have a total of 1 - 2 peptide sequences identified in the protein group and each of those peptides were unique to the particular protein. The isolation interference is quite low in all the proteins except for mihF and hup, which have a percentage of 86 and average of 42.5 respectively. Due to TMT labelling and certain proteins, for example mihF and hup, having similarities with others, the interference was raised. Percolator increases yield and assigns reliable confidence measures such as q-value and PEP. Q-value finds the false positives in the data and PEP finds the probability that the observed PSM is incorrect and is instead random. Both statistics have found that all proteins had a q-value of less than 0.006 and a PEP of less than 0.04, considering the data highly confident with a very low chance it is incorrect.
