# Supplementary material for: A meta-proteomics approach using autopsy material from the pre-antibiotic era from patients with untreated pulmonary tuberculosis to identify proteins present in early lesions of post-primary tuberculosis
Source: PLoS One. 2026 Apr 1;21(4):e0345052. doi: 10.1371/journal.pone.0345052 (PMC13042780; doi:10.1371/journal.pone.0345052)
Supplement: S1 File — (A) Processing workflow used to map MS/MS spectra to the curated FASTA database, including peptide-spectrum matching, target/decoy strategy, and false discovery rate (FDR) filtering to assign peptide and protein confidence. (B) Consensus workflow used for reporter-ion quantification, normalization, and scaling across samples. Key workflow parameters are provided in the supplementary methods section. S2 Fig. Manual validation of bacterial peptide identification using MS1 precursor isotope pattern and MS2 fragmentation. Representative MS1 precursor isotope pattern illustrating manual interrogation of mass spectra for bacterial proteins. Multiple candidate peptide matches are shown; the peptide selected by Proteome Discoverer as the highest-confidence match (highlighted in yellow) was subsequently fragmented for MS2 confirmation. The y-axis indicates signal intensity (×10³), and the x-axis indicates mass-to-charge ratio (m/z). Spectra were visualized using Thermo Proteome Discoverer v2.5. S3 Fig. Precursor isotope patterns and interference levels for mycobacterial peptide identifications across samples. MS1 precursor isotope patterns for the peptides used to identify mycobacterial proteins in each sample. In all cases, a single peptide supported protein identification. Sample P71658 showed the highest isolation interference, consistent with multiple potential peptides matches; however, Proteome Discoverer resolved a single high-confidence peptide identification. S1 Table. Curated FASTA database composition used for metaproteomic MS/MS searches. List of species included in the custom reference database used for peptide-spectrum matching, including organism name, UniProt proteome identifier, number of proteins (proteomes) included, and rationale for inclusion based on known associations with TB pathology and pulmonary comorbidities. S2 Table. Total number of proteins identified per sample. Total protein identifications obtained per sample after database search and filter [file pone.0345052.s001.zip › S1_Fig.docx]

**Supplementary Figure 1. Proteome Discoverer workflow and key settings used for peptide identification and TMT-based quantification.** (A) Processing workflow used to map MS/MS spectra to the curated FASTA database, including peptide-spectrum matching, target/decoy strategy, and false discovery rate (FDR) filtering to assign peptide and protein confidence. (B) Consensus workflow used for reporter-ion quantification, normalization, and scaling across samples. Key workflow parameters are provided in the supplementary methods section.

A.
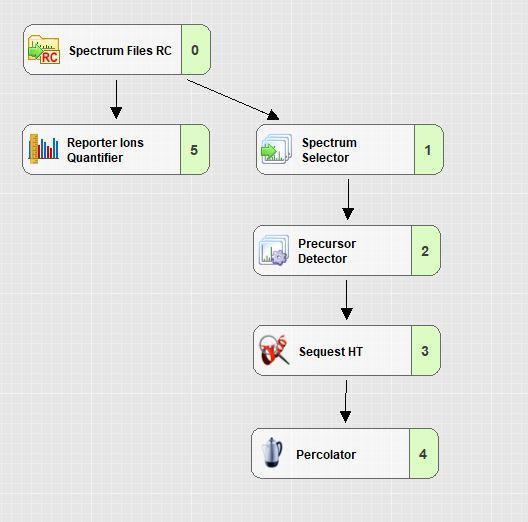
B.
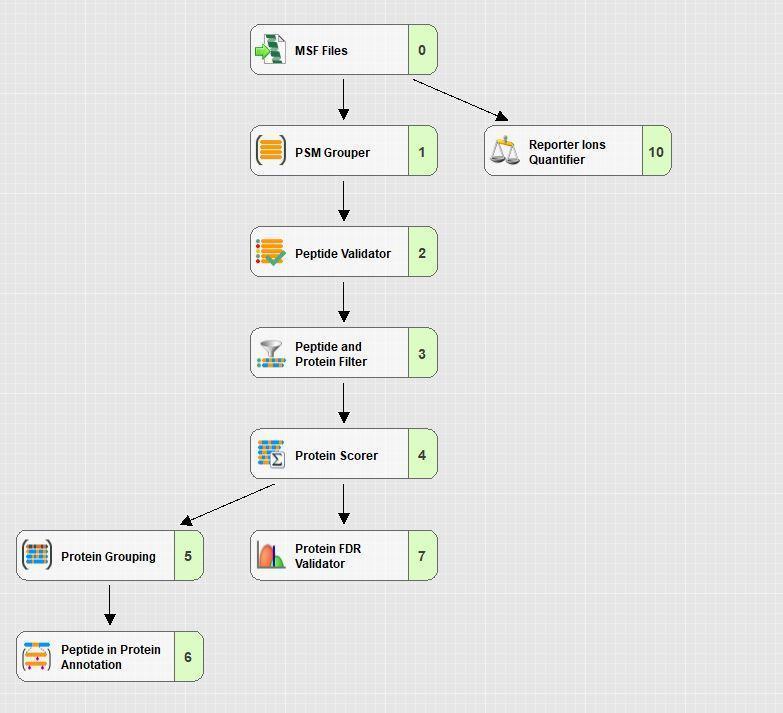


Supplement: Consensus Method for Thermo Proteome Discoverer

Result name: Beatrice_F

Result file: D:\Even\Beatrice_Normann\Tuberculosis first search22022022\Beatrice_F-(2).pd Result

Description: Result filtered for high confident peptides, with enhanced peptide and protein annotations. Add FASTA file with common contaminants to the Protein Marker node. Quan abundances are normalized to the same total peptide amount per channel and scaled, so that the average a bunce per protein and peptide is 100.

Workflow based on template: CWF_Comprehensive_Enhanced Annotation_Reporter_Quan

Creation date: 3/2/2022 10:10:05 AM

Created with Discoverer version: 2.5.0.400

------------------------------------------------------------------

The workflow tree:

------------------------------------------------------------------

|-(0) MSF Files

|-(1) PSM Grouper

|-(2) Peptide Validator

|-(3) Peptide and Protein Filter

|-(4) Protein Scorer

|-(7) Protein FDR Validator

|-(5) Protein Grouping

|-(6) Peptide in Protein Annotation

|-(9) Protein Marker

|-(10) Reporter Ions Quantifier

Post-processing nodes:

--------------------------------

|-(11) Result Statistics

|-(12) Display Settings

|-(13) Data Distributions

------------------------------------------------------------------

Processing node 0: MSF Files

------------------------------------------------------------------

1. Storage Settings:

- Spectra to Store: Identified or Quantified

- Feature Traces to Store: All

2. Merging of Identified Peptide and Proteins:

- Merge Mode: Globally by Search Engine Type

3. FASTA Title Line Display:

- Reported FASTA Title Lines: Best match

- Title Line Rule: standard

4. PSM Filters:

- Maximum Delta Cn: 0.05

- Maximum Rank: 0

- Maximum Delta Mass: 0 ppm

Hidden Parameters:

- MSF File(s): D:\Even\Beatrice_Normann\Tuberculosis first search22022022\Beatrice_F-(2).msf

------------------------------------------------------------------

Processing node 1: PSM Grouper

------------------------------------------------------------------

1. Peptide Group Modifications:

- Site Probability Threshold: 75

------------------------------------------------------------------

Processing node 2: Peptide Validator

------------------------------------------------------------------

1. General Validation Settings:

- Validation Mode: Automatic (Control peptide level error rate if possible)

- Target FDR (Strict) for PSMs: 0.01

- Target FDR (Relaxed) for PSMs: 0.05

- Target FDR (Strict) for Peptides: 0.01

- Target FDR (Relaxed) for Peptides: 0.05

2. Specific Validation Settings:

- Validation Based on q-Value

- Target/Decoy Selection for PSM Level FDR Calculation Based on Score: Automatic

- Reset Confidences for Nodes without Decoy Search (Fixed score thresholds): False

------------------------------------------------------------------

Processing node 3: Peptide and Protein Filter

------------------------------------------------------------------

1. Peptide Filters:

- Peptide Confidence At Least: High

- Keep Lower Confident PSMs: False

- Minimum Peptide Length: 6

- Remove Peptides Without Protein Reference: False

2. Protein Filters:

- Minimum Number of Peptide Sequences: 1

- Count Only Rank 1 Peptides: False

- Count Peptides Only for Top Scored Protein: False

------------------------------------------------------------------

Processing node 4: Protein Scorer

------------------------------------------------------------------

No parameters

------------------------------------------------------------------

Processing node 7: Protein FDR Validator

------------------------------------------------------------------

1. Confidence Thresholds:

- Target FDR (Strict): 0.01

- Target FDR (Relaxed): 0.05

------------------------------------------------------------------

Processing node 5: Protein Grouping

------------------------------------------------------------------

1. Protein Grouping:

- Apply strict parsimony principle: True

------------------------------------------------------------------

Processing node 6: Peptide in Protein Annotation

------------------------------------------------------------------

1. Flanking Residues:

- Annotate Flanking Residues of the Peptide: True

- Number Flanking Residues in Connection Tables: 1

2. Modifications in Peptide:

- Protein Modifications Reported: Only for Master Proteins

3. Modifications in Protein:

- Modification Sites Reported: All and Specific

- Minimum PSM Confidence: High

- Report Only PTMs: True

4. Positions in Protein:

- Protein Positions for Peptides: Only for Master Proteins

------------------------------------------------------------------

Processing node 9: Protein Marker

------------------------------------------------------------------

1. Contaminant Database:

- Protein Database: Contaminants.fasta

2. Additional Marker Database:

- Protein Database: 020982_7252uniprot-mycobacterium+tuberculosis-filtered-reviewed yes.fasta

5. Annotate Species:

- As Species Map: False

- As Species Names: False

6. Mark Additional Entities:

- Annotation Groups: False

- Pathway Groups: False

- Modification Sites: True

- Peptide Isoform Groups: True

------------------------------------------------------------------

Processing node 10: Reporter Ions Quantifier

------------------------------------------------------------------

1. General Quantification Settings:

- Peptides to Use: Unique + Razor

- Consider Protein Groups for Peptide Uniqueness: True

- Use Shared Quan Results: True

- Reject Quan Results with Missing Channels: False

2. Reporter Quantification:

- Reporter Abundance Based On: Intensity

- Apply Quan Value Corrections: True

- Co-Isolation Threshold: 50

- Average Reporter S/N Threshold: 10

- SPS Mass Matches [%] Threshold: 65

- Minimum Channel Occupancy [%] Threshold: 0

3. Normalization and Scaling:

- Normalization Mode: Total Peptide Amount

- Scaling Mode: None

4. Exclude Peptides from Protein Quantification:

- For Normalization: Use All Peptides

- For Protein Roll-Up: Use All Peptides

- For Pairwise Ratios: Exclude Modified

5. Quan Rollup and Hypothesis Testing:

- Protein Ratio Calculation: Protein Abundance Based

- Maximum Allowed Fold Change: 100

- Imputation Mode: None

- Hypothesis Test: ANOVA (Individual Proteins)

6. Quan Ratio Distributions:

- 1st Fold Change Threshold: 2

- 2nd Fold Change Threshold: 4

- 3rd Fold Change Threshold: 6

- 4th Fold Change Threshold: 8

- 5th Fold Change Threshold: 10

------------------------------------------------------------------

Processing node 11: Result Statistics

------------------------------------------------------------------

No parameters

------------------------------------------------------------------

Processing node 12: Display Settings

------------------------------------------------------------------

1. General:

- Filter Set:

### Filter Set MasterProteinFilter.filterset contains the following filters:

### Row Filter for TargetProtein:

### Master is equal to Master

###

'magellan filter set' 1 'MasterProteinFilter.filterset' FiltersetProperties 1 'LastFileName' 'C:\Users\frank.berg\Desktop\MasterProteinFilter.filterset' Filter 'TargetProtein' 1 NARY_AND 1 = FilterConditionProperties 1 'NamedComparableFilterCondition/DisplayPropertyHint' 'Master' property 'Thermo.PD.EntityDataFramework.MasterProteinAssessment, Thermo.Magellan.EntityDataFramework' 'IsMasterProtein' constant 'Thermo.PD.EntityDataFramework.MasterProteinAssessment, Thermo.Magellan.EntityDataFramework' 'IsMasterProtein'

------------------------------------------------------------------

Processing node 13: Data Distributions

------------------------------------------------------------------

1. ID Distributions (Bottom-up):

- Peptides to Use: Only unique peptides based on protein groups
