# Supplementary material for: A meta-proteomics approach using autopsy material from the pre-antibiotic era from patients with untreated pulmonary tuberculosis to identify proteins present in early lesions of post-primary tuberculosis
Source: PLoS One. 2026 Apr 1;21(4):e0345052. doi: 10.1371/journal.pone.0345052 (PMC13042780; doi:10.1371/journal.pone.0345052)

**Supplementary Figure 3. Precursor isotope patterns and interference levels for mycobacterial peptide identifications across samples.** MS1 precursor isotope patterns for the peptides used to identify mycobacterial proteins in each sample. In all cases, a single peptide supported protein identification. Sample P71658 showed the highest isolation interference, consistent with multiple potential peptides matches; however, Proteome Discoverer resolved a single high-confidence peptide identification.


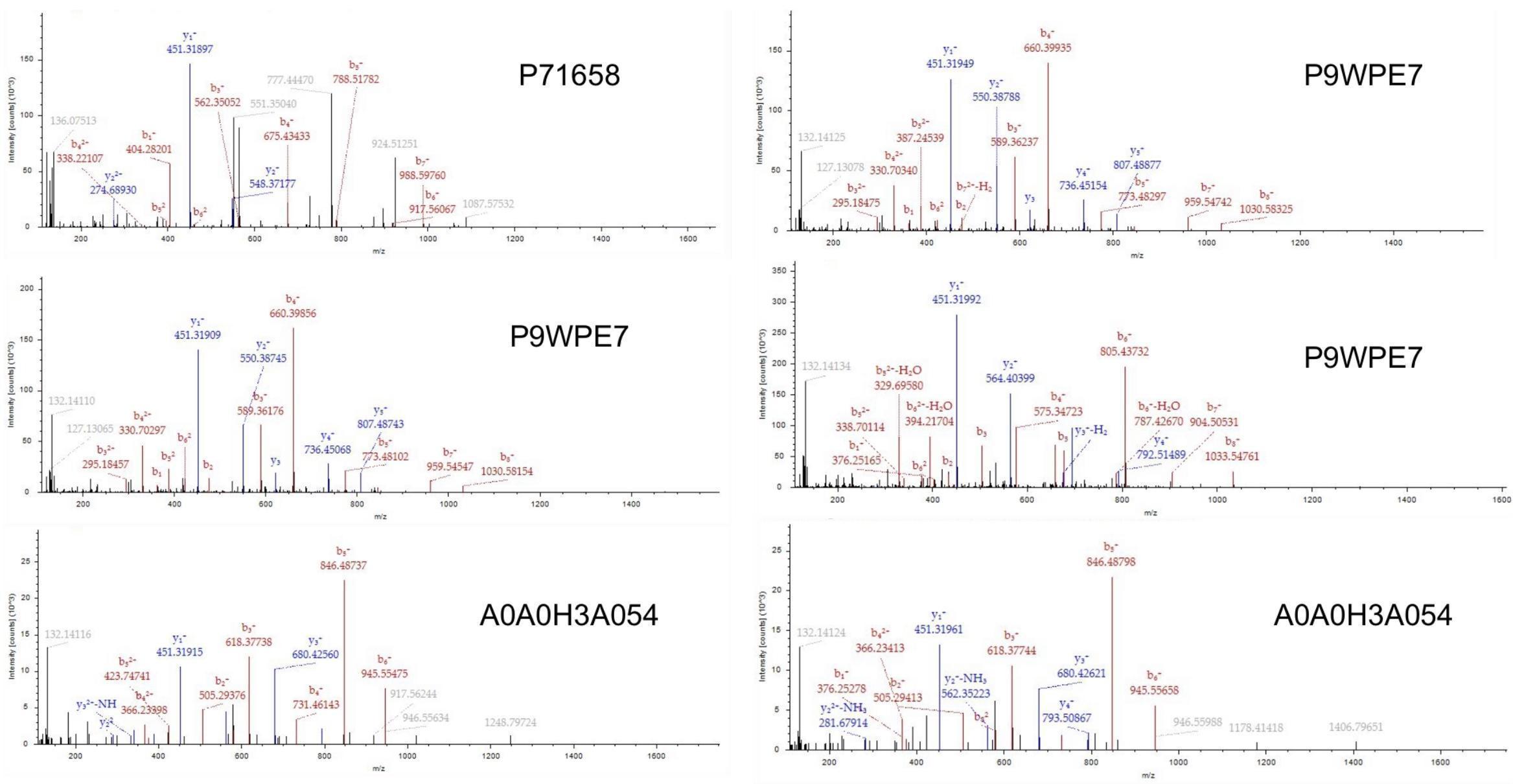

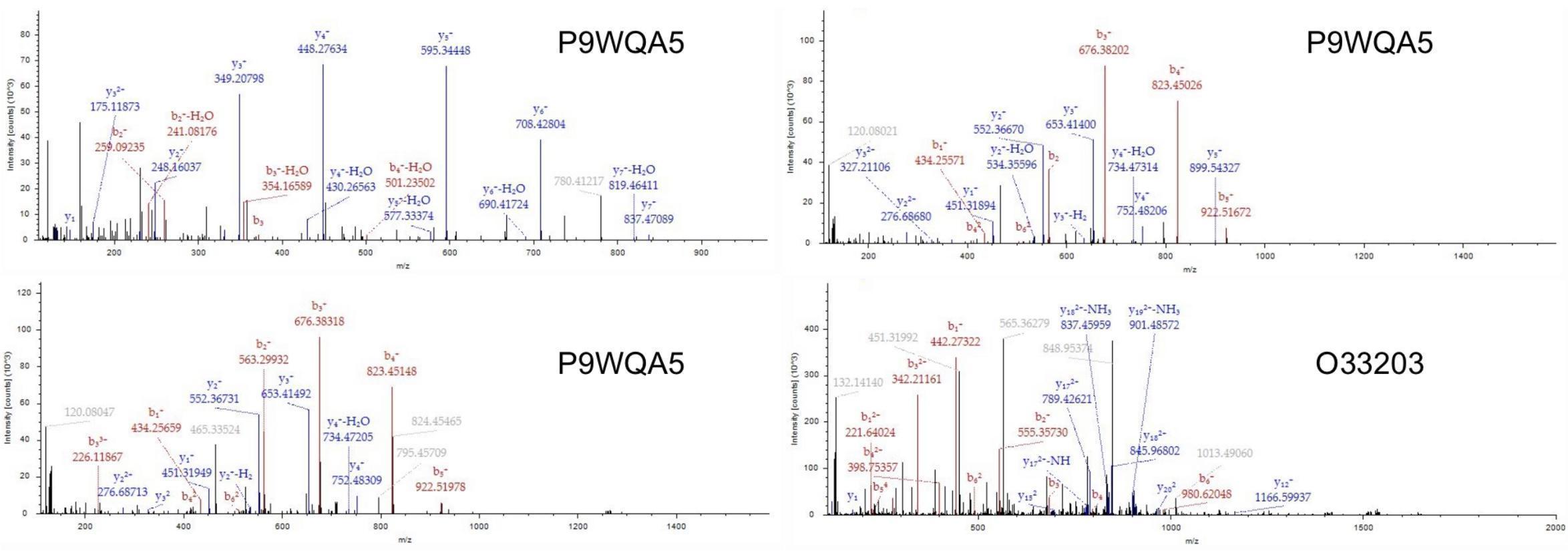

Supplement: S1 File — (A) Processing workflow used to map MS/MS spectra to the curated FASTA database, including peptide-spectrum matching, target/decoy strategy, and false discovery rate (FDR) filtering to assign peptide and protein confidence. (B) Consensus workflow used for reporter-ion quantification, normalization, and scaling across samples. Key workflow parameters are provided in the supplementary methods section. S2 Fig. Manual validation of bacterial peptide identification using MS1 precursor isotope pattern and MS2 fragmentation. Representative MS1 precursor isotope pattern illustrating manual interrogation of mass spectra for bacterial proteins. Multiple candidate peptide matches are shown; the peptide selected by Proteome Discoverer as the highest-confidence match (highlighted in yellow) was subsequently fragmented for MS2 confirmation. The y-axis indicates signal intensity (×10³), and the x-axis indicates mass-to-charge ratio (m/z). Spectra were visualized using Thermo Proteome Discoverer v2.5. S3 Fig. Precursor isotope patterns and interference levels for mycobacterial peptide identifications across samples. MS1 precursor isotope patterns for the peptides used to identify mycobacterial proteins in each sample. In all cases, a single peptide supported protein identification. Sample P71658 showed the highest isolation interference, consistent with multiple potential peptides matches; however, Proteome Discoverer resolved a single high-confidence peptide identification. S1 Table. Curated FASTA database composition used for metaproteomic MS/MS searches. List of species included in the custom reference database used for peptide-spectrum matching, including organism name, UniProt proteome identifier, number of proteins (proteomes) included, and rationale for inclusion based on known associations with TB pathology and pulmonary comorbidities. S2 Table. Total number of proteins identified per sample. Total protein identifications obtained per sample after database search and filter [file pone.0345052.s001.zip › S3_Fig.docx]
